# Role of Polyinosinic:Polycytidylic Acid-Induced Maternal Immune Activation and Subsequent Immune Challenge in the Behaviour and Microglial Cell Trajectory in Adult Offspring: A Study of the Neurodevelopmental Model of Schizophrenia

**DOI:** 10.3390/ijms22041558

**Published:** 2021-02-04

**Authors:** Katarzyna Chamera, Ewa Trojan, Katarzyna Kotarska, Magdalena Szuster-Głuszczak, Natalia Bryniarska, Kinga Tylek, Agnieszka Basta-Kaim

**Affiliations:** Laboratory of Immunoendocrinology, Department of Experimental Neuroendocrinology, Maj Institute of Pharmacology, Polish Academy of Sciences, 12 Smętna St., 31-343 Kraków, Poland; chamera@if-pan.krakow.pl (K.C.); trojan@if-pan.krakow.pl (E.T.); katarzyna.kotarska1@gmail.com (K.K.); szuster@if-pan.krakow.pl (M.S.-G.); natbry@if-pan.krakow.pl (N.B.); tylek@if-pan.krakow.pl (K.T.)

**Keywords:** maternal immune activation, polyinosinic:polycytidylic acid, CX3CL1-CX3CR1, CD200-CD200R, microglial reactivity, two-hit hypothesis of schizophrenia, behavioural disturbances

## Abstract

Multiple lines of evidence support the pathogenic role of maternal immune activation (MIA) in the occurrence of the schizophrenia-like disturbances in offspring. While in the brain the homeostatic role of neuron-microglia protein systems is well documented, the participation of the CX3CL1-CX3CR1 and CD200-CD200R dyads in the adverse impact of MIA often goes under-recognized. Therefore, in the present study, we examined the effect of MIA induced by polyinosinic:polycytidylic acid (Poly I:C) on the CX3CL1-CX3CR1 and CD200-CD200R axes, microglial trajectory (*MhcII*, *Cd40*, *iNos*, *Il-1β*, *Tnf-α*, *Il-6*, *Arg1*, *Igf-1*, *Tgf-β* and *Il-4*), and schizophrenia-like behaviour in adult male offspring of Sprague-Dawley rats. Additionally, according to the “two-hit” hypothesis of schizophrenia, we evaluated the influence of acute challenge with Poly I:C in adult prenatally MIA-exposed animals on the above parameters. In the present study, MIA evoked by Poly I:C injection in the late period of gestation led to the appearance of schizophrenia-like disturbances in adult offspring. Our results revealed the deficits manifested as a diminished number of aggressive interactions, presence of depressive-like episodes, and increase of exploratory activity, as well as a dichotomy in the sensorimotor gating in the prepulse inhibition (PPI) test expressed as two behavioural phenotypes (MIA_PPI-low_ and MIA_PPI-high_). Furthermore, in the offspring rats subjected to a prenatal challenge (i.e., MIA) we noticed the lack of modulation of behavioural changes after the additional acute immune stimulus (Poly I:C) in adulthood. The important finding reported in this article is that MIA affects the expression and levels of the neuron-microglia proteins in the frontal cortex and hippocampus of adult offspring. We found that the changes in the CX3CL1-CX3CR1 axis could affect microglial trajectory, including decreased hippocampal mRNA level of *MhcII* and elevated cortical expression of *Igf-1* in the MIA_PPI-high_ animals and/or could cause the up-regulation of an inflammatory response (*Il-6*, *Tnf-α*, *iNos*) after the “second hit” in both examined brain regions and, at least in part, might differentiate behavioural disturbances in adult offspring. Consequently, the future effort to identify the biological background of these interactions in the Poly I:C-induced MIA model in Sprague-Dawley rats is desirable to unequivocally clarify this issue.

## 1. Introduction

Multiple lines of evidence suggest that the aetiology of schizophrenia involves aberrant neurodevelopmental processes [1,2,3]. The onset of the schizophrenic full-blown psychotic state usually occurs in late adolescence or early adulthood as an outcome of pathological processes arising during early brain development, long before the illness is clinically expressed [4,5]. Many studies have demonstrated a link between maternal immune activation (MIA) following infections with viruses, such as influenza, rubella, herpes simplex, or cytomegalovirus [6,7], and schizophrenic disturbances in offspring [8]. Preclinical support for these epidemiological associations has also been strengthened by translational rodent models showing brain and behavioural disturbances following prenatal exposure to MIA [9,10,11].

The synthetic double-stranded RNA mimetic polyinosinic:polycytidylic acid (Poly I:C) has been used to generate a standard model of viral infections. Researchers have shown that this model captures a wide spectrum of behavioural schizophrenia-like abnormalities, including disturbances in prepulse inhibition (PPI) and cognition [12], exaggerated locomotor activity [13], deficiency in learning skills [14], dysregulation of neurotransmission [15], and brain morphological abnormalities [16,17]. During Poly I:C-induced MIA, elevated maternal serum levels of cytokines, including IL-6, were found to be critical for the development of neurological deficits in offspring [18,19]. However, in some cases, the manifestation of these symptoms occurs only after the application of the second trigger in adulthood, which is widely postulated by the “two-hit” hypothesis of schizophrenia [20,21]. 

Recently, there has been growing evidence that the pathophysiology of schizophrenia may involve aberrant microglial function and related immune and behavioural changes [22]. Positron emission computed tomography and postmortem studies have demonstrated microglia overactivation in schizophrenic patients, particularly in the course of the acute psychotic phase [23,24], as well as in subjects with high risk for developing this psychosis [25].

In the brain, microglial cells are known to control several developmental processes [26,27] and an innate immune response by the induction, propagation, and resolution of inflammatory signals [28,29,30]. Under basal conditions, microglia examine their surroundings and maintain the ability to respond highly sensitively to potential challenges to homeostasis [31]. When activated, microglia release cytokines and chemokines, engage in phagocytosis, secrete growth factors [32], and regulate astrocyte pathogenic activity [33]. Considering the dual role of microglia, most often two phenotypes of these cells are distinguished: (1) proinflammatory, which is characterized by the expression of MHCII, CD40, iNOS, IL-1β, TNF-α, and IL-6; and (2) alternative, which is considered anti-inflammatory and defined through the production of ARG1, IGF-1, TGF-β, IL-4, and IL-10 [34]. Strict regulation of microglial activation is necessary to limit the effects of harmful factors and to maintain only a transient neuroinflammatory response.

One of the crucial mechanisms involved in the regulation of microglial reactivity is based on proper communication between neurons and microglia. This interaction is executed mainly via the specialized, endogenous protein systems CX3CL1-CX3CR1 and CD200-CD200R, which represent unique ligand-receptor axes [35,36,37]. CX3CL1 is a chemokine whose expression is remarkably higher in the brain than in the periphery [38], and neurons are its main source. This protein is the only ligand for CX3CR1 present on microglial cells. Apart from the role of CX3CL1 in the induction of chemotaxis and cell adhesion, the CX3CL1-CX3CR1 dyad also regulates neuronal survival [39], maintenance of synaptic plasticity [40], activation and proper functioning of microglia [41], production of inflammatory factors and the resolution of inflammation [42,43]. CD200 is a surface antigen with immunosuppressive properties that maintains the resting state of microglial cells [44]. This membrane glycoprotein is expressed ubiquitously on neurons, endothelial cells, and oligodendrocytes [45,46,47] and exerts its biological effect through the cognate receptor of CD200 (CD200R), which is present almost exclusively on myeloid cells, including microglia. The recognized function of the CD200-CD200R pathway consists of the suppression of the proinflammatory response of microglia to immune stimuli [44], which, if prolonged, may lead to an exacerbated inflammatory reaction and neurodegeneration [48,49,50,51]. Additionally, the CD200-CD200R system plays a regulatory role in the brain by affecting the proliferation and apoptosis of microglial cells [52]. 

In this proof-of-concept study, we explored the hypothesis that acute treatment with Poly I:C in the late period of gestation modulates the CX3CL1-CX3CR1 and/or CD200-CD200R axes and microglial trajectory and entails schizophrenia-like deficits in adult male offspring of Sprague-Dawley rats. To achieve this goal, we explored the impact of MIA on various behavioural parameters using a range of tests capable of assessing positive, negative, schizoaffective, and anxiety-related symptoms in adult offspring exposed additionally to an immune challenge in adulthood. Within this focus, we further measured the gene and protein expression of the CX3CL1-CX3CR1 and CD200-CD200R pathways, and we explored the potential role of the dual microglial phenotype in the context of the “two-hit” hypothesis of schizophrenia. The biochemical analyses were performed in the frontal cortex and hippocampus since morphological and functional changes in these regions are responsible for the occurrence and severity of specific symptoms of schizophrenia [53,54,55].

## 2. Results

### 2.1. Light-Dark Box Test

**Figure 1:**
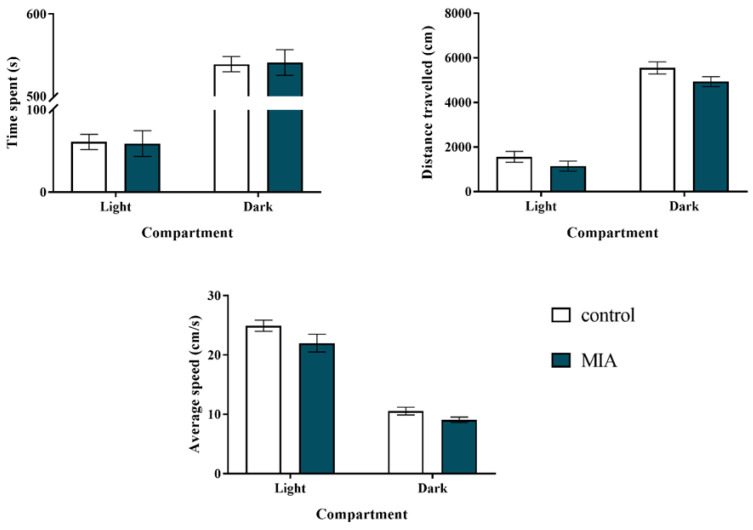
Impact of maternal immune activation (MIA) on anxiety-like behaviours of adult male Sprague-Dawley offspring, measured in the light-dark box test. *n* = 28 in the control group, *n* = 43 in the MIA group. The results are presented as the means ± standard errors of the mean (SEM).

We conducted a light-dark box test to assess the effect of MIA on anxiety-like behaviour in offspring at postnatal day 90 (PND90). As shown in Figure 1, the MIA group did not differ from the control animals in terms of all parameters (time spent, distance travelled, and average speed) measured either in the light or dark compartment of the experimental apparatus.

### 2.2. Social Interaction Test

Schizophrenia highly impairs the social functioning of patients and inevitably leads to social withdrawal that often persists despite treatment [56,57]. We showed that MIA did not induce alterations either in the time or the number of non-aggressive behaviours; however, it decreased the time and the number of aggressive activities of the male offspring at PND90, thereby limiting this type of interaction (Table 1).

### 2.3. Forced Swim Test

**Figure 2:**
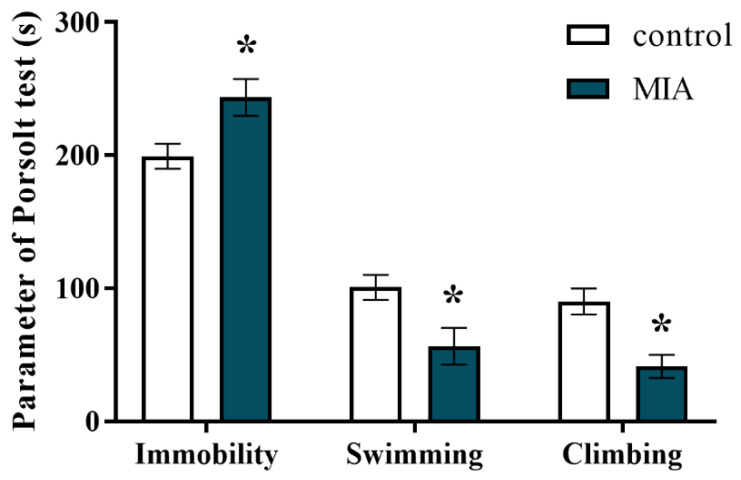
Impact of maternal immune activation (MIA) on depressive-like behaviour of adult male Sprague-Dawley offspring, measured in the forced swim test (FST, Porsolt test). *n* = 8 in each group. Immobility, swimming, and climbing times (in seconds) are presented as the means ± standard errors of the mean (SEM). * *p* < 0.05 vs. control group.

Some behavioural disturbances are reported both for individuals with schizophrenia and schizoaffective disorder [58,59]. In the present study, we applied the forced swim test (FST, Porsolt test) to examine whether the animals from the MIA group displayed depressive-like behaviour. The obtained results revealed that MIA resulted in significantly prolonged immobility time, and thereby shortened the swimming and climbing times in the offspring at PND95 (Figure 2). Therefore, MIA with Poly I:C induced depressive-like changes in adult male rats.

### 2.4. Exploratory Activity

**Figure 3:**
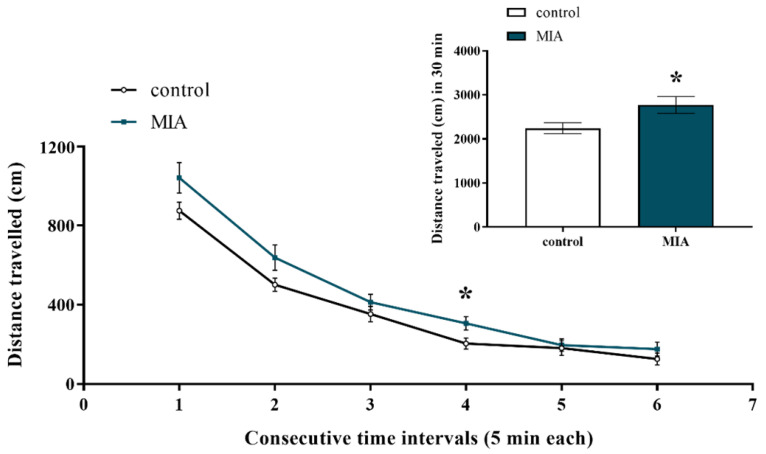
Impact of maternal immune activation (MIA) on the exploratory activity of adult male Sprague-Dawley offspring. The exploratory activity is expressed as the distance travelled (in cm) in respective time intervals (5 min each) and the total distance travelled during 30-minute-interval (shown on the inset). *n* = 13 in the control group, *n* = 14 in the MIA group. The results are presented as the means ± standard errors of the mean (SEM). * *p* < 0.05 vs. control group.

Under basal conditions, exploratory behaviour allows animals to collect information about unfamiliar parts of an environment [60]. Hyperactivity, however, is a schizophrenia-like feature in animal models that resembles psychotic symptoms in patients [61]. In our study, we examined the effect of MIA on the exploratory activity of male offspring at PND88. Statistical analysis showed that rats from the MIA group were more active than the control animals as evidenced by an increase in the total distance travelled (Figure 3). The exploration of the MIA offspring was particularly enhanced during the fourth interval of the experiment (Figure 3). Hence, MIA affected novelty-related behaviour, leading to the occurrence of hyperactivity symptoms in the offspring.

### 2.5. Prepulse Inhibition of the Acoustic Startle Response

PPI refers to the phenomenon where a weak prestimulus transiently inhibits the response closely following a strong sensory stimulus [58]. Deficient sensorimotor gating (measured using PPI) has been observed in patients [62,63,64] and animal models [21,65,66] of schizophrenia. Initially, we investigated the effect of MIA on the PPI in rats at two time points: at PND30 and PND60 (Table 2). The results revealed that at these stages of the offspring’s life, MIA did not influence the PPI response of the animals.

**Figure 4:**
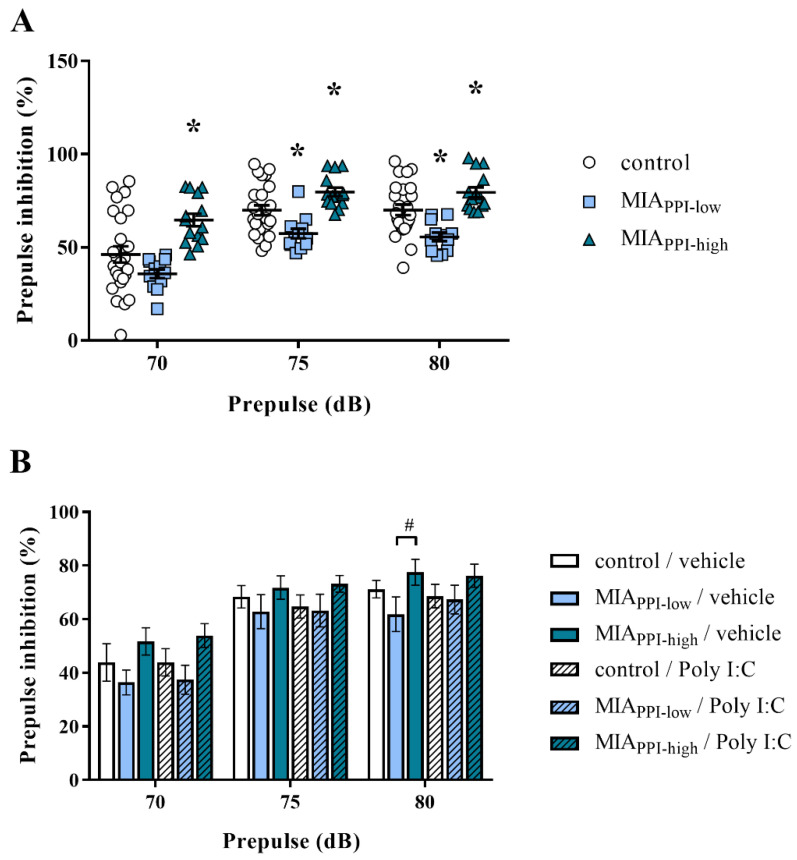
(**A**) Prepulse inhibition (PPI) test at postnatal day 100 (PND100) revealed two distinct behavioural phenotypes: MIA_PPI-low_ (with the deficit in PPI) and MIA_PPI-high_ (without the deficit) in male Sprague-Dawley offspring exposed to maternal immune activation (MIA). *n* = 25 in the control group, *n* = 12 in the MIA_PPI-low_ group, *n* = 14 in the MIA_PPI-high_ group. * *p* < 0.05 vs. control group. The results are presented as the individual data points of the percentage of PPI (PPI%) induced by each prepulse intensity with the means ± standard errors of the mean (SEM). Data were calculated based on the average startle amplitudes (AVGs). (**B**) At PND120, the animals were additionally subjected to acute challenge with polyinosinic:polycytidylic acid (Poly I:C) and 2 h later, the PPI was evaluated again. *n* = 6–10. # *p* < 0.05 vs. MIA_PPI-low_ + vehicle. The results are presented as the means of PPI% induced by each prepulse intensity ± SEM. Data were calculated based on AVGs.

Interestingly, when the PPI was examined at PND100, we observed that MIA caused an occurrence of two response patterns, based on which the offspring were divided into the following categories: MIA_PPI-low_ (characterized by the deficit in PPI) and MIA_PPI-high_ (without the deficit in PPI) (Figure 4A). In more detail, the MIA_PPI-low_ group displayed inhibition of sensorimotor gating compared to the control animals for the 75 dB and 80 dB prepulse intensities. The MIA_PPI-high_ rats were characterized by an increase in the PPI compared to the control offspring for all analysed prepulse levels: 70 dB, 75 dB, and 80 dB (Figure 4A).

At PND120, the animals were subjected to the “second hit” with Poly I:C to establish whether the acute challenge in adulthood could further alter the PPI in the male offspring of dams after MIA (Figure 4B). The additional immune stimulation did not affect sensorimotor gating in any of the analysed groups, resulting in insufficient triggering behavioural deficits even in the rats prenatally exposed to MIA.

### 2.6. mRNA Expression of Microglial Markers in the Frontal Cortices and Hippocampi of Adult Male Offspring

**Figure 5:**
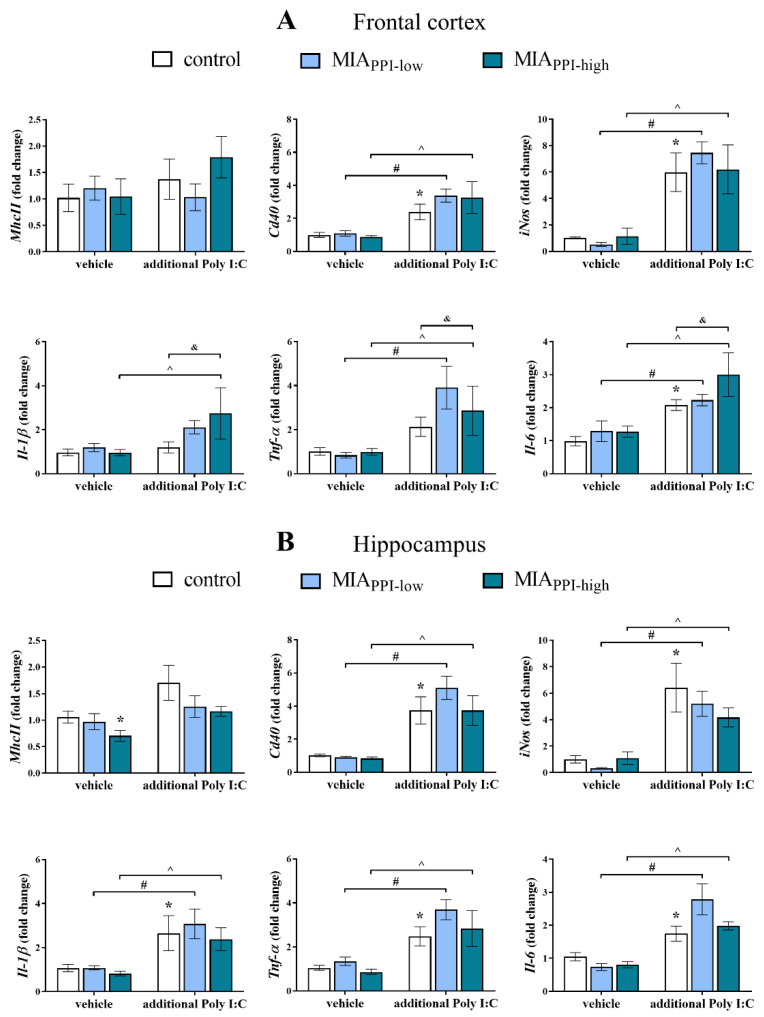
Impact of maternal immune activation (MIA) and the additional acute challenge with polyinosinic:polycytidylic acid (Poly I:C) on the gene expression of the proinflammatory microglial markers: *MhcII*, *Cd40*, *iNos*, *Il-1β*, *Tnf-α,* and *Il-6* in the frontal cortices (**A**) and hippocampi (**B**) of male Sprague-Dawley offspring at postnatal day 120 (PND120). The mRNA levels were measured using qRT-PCR with *n* = up to 9 in each group. The results are presented as the average fold change ± standard errors of the mean (SEM). * *p* < 0.05 vs. control + vehicle, # *p* < 0.05 vs. MIA_PPI-low_ + vehicle, ^ *p* < 0.05 vs. MIA_PPI-high_ + vehicle, & *p* < 0.05 vs. control + Poly I:C.

In the first set of biochemical experiments, we determined the mRNA expression of pro- (*MhcII*, *Cd40*, *iNos*, *Il-1β*, *Tnf-α,* and *Il-6*) and anti-inflammatory (*Arg1*, *Igf-1*, *Tgf-β,* and *Il-4*) factors that are considered microglial markers. As shown in Figure 5A, MIA did not affect the mRNA levels of any of the analysed proinflammatory factors in the frontal cortices of the MIA_PPI-low_ or MIA_PPI-high_ offspring.

The “second hit” with Poly I:C up-regulated the expression of *Cd40*, *iNos,* and *Il-6* in the frontal cortices of the control group (Figure 5A). Moreover, acute treatment with Poly I:C in adulthood increased the cortical expression of *Cd40*, *iNos*, *Tnf-α,* and *Il-6* in the MIA_PPI-low_ rats. The impact of the additional injection of Poly I:C was also observed in the MIA_PPI-high_ animals, where elevated mRNA levels of *Cd40*, *iNos*, *Il-1β*, *Tnf-α,* and *Il-6* in the frontal cortex were detected. The changes in the MIA_PPI-high_ offspring that received Poly I:C in adulthood were more distinct than those in the control animals at the levels of *Il-1β*, *Tnf-α,* and *Il-6* (Figure 5A).

**Figure 6:**
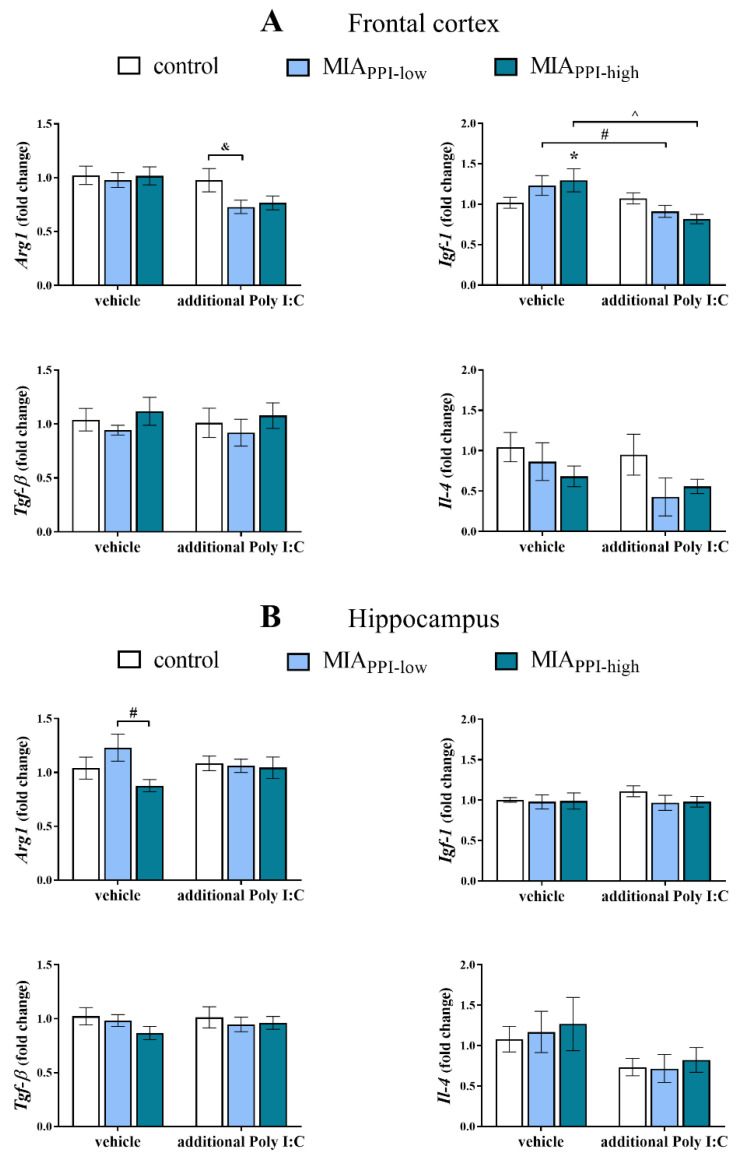
Impact of maternal immune activation (MIA) and the additional acute challenge with polyinosinic:polycytidylic acid (Poly I:C) on the gene expression of the anti-inflammatory microglial markers *Arg1*, *Igf-1*, *Tgf-β,* and *Il-4* in the frontal cortices (**A**) and hippocampi (**B**) of male Sprague-Dawley offspring at postnatal day 120 (PND120). The mRNA levels were measured using qRT-PCR with *n* = 4–9 in each group. The results are presented as the average fold change ± standard errors of the mean (SEM). * *p* < 0.05 vs. control + vehicle, # *p* < 0.05 vs. MIA_PPI-low_ + vehicle, ^ *p* < 0.05 vs. MIA_PPI-high_ + vehicle, & *p* < 0.05 vs. control + Poly I:C.

On the other hand, we demonstrated that in the frontal cortex, the effect of MIA on the anti-inflammatory parameters was less pronounced (Figure 6A). Among the tested markers, we only observed that in the MIA_PPI-high_ offspring, the mRNA level of *Igf-1* was significantly higher than that in the control animals (Figure 6A). The additional stimulation with Poly I:C decreased the expression of *Igf-1* in both the MIA_PPI-low_ and MIA_PPI-high_ offspring. Simultaneously, the cortical level of *Arg1* in the MIA_PPI-low_ rats after the “second hit” with Poly I:C was lower than that in the control group (Figure 6A).

Regarding the results obtained from the hippocampus, we observed that MIA reduced the mRNA level of *MhcII* in the MIA_PPI-high_ animals (Figure 5B).

The injection of Poly I:C in adulthood enhanced the expression of *Cd40*, *iNos*, *Il-1β*, *Tnf-α,* and *Il-6* in the hippocampi of the control animals (Figure 5B).

Stimulation with Poly I:C in adulthood increased the expression of the vast majority of the proinflammatory microglial phenotype markers in the frontal cortices of the MIA_PPI-low_ offspring: *Cd40*, *iNos*, *Il-1β*, *Tnf-α,* and *Il-6* (Figure 5B). A similar effect was demonstrated for the MIA_PPI-high_ rats, as evidenced by the increased hippocampal expression of *Cd40*, *iNos, Il-1β*, *Tnf-α*, and *Il-6* (Figure 5B).

In the case of anti-inflammatory factors, statistical analysis revealed only reduced expression of *Arg1* in the hippocampi of the MIA_PPI-high_ offspring when compared to the MIA_PPI-low_ animals (Figure 6B). No effect of the “second hit” with Poly I:C on the hippocampal levels of the anti-inflammatory markers was observed in any of the investigated groups (Figure 6B).

### 2.7. mRNA Expression of Cx3cl1, Cx3cr1, Cd200 and Cd200r in the Frontal Cortices and Hippocampi of Adult Male Offspring

Considering the alterations in the pro- and anti-inflammatory factors, we examined the effect of MIA and the acute systemic injection of Poly I:C in adulthood on the mRNA levels of neuronal ligands (*Cx3cl1*, *Cd200*) and their corresponding microglial receptors (*Cx3cr1*, *Cd200r*) in the frontal cortices and hippocampi of male offspring (Table 3). The cortical expression of the analysed signalling pathways was not affected by MIA. The additional stimulation with Poly I:C decreased the mRNA level of *Cx3cl1* in the frontal cortex of the MIA_PPI-low_ animals compared to the control rats. Regarding the expression of *Cx3cr1*, the impact of the “second hit” was present in the frontal cortices of the MIA_PPI-high_ offspring (Table 3).

Analyses of samples obtained from the hippocampi of the MIA_PPI-high_ animals revealed that MIA affected the *Cx3cl1-Cx3cr1* system, as evidenced by a decline in the mRNA levels of *Cx3cl1* and *Cx3cr1* (Table 3). The expression of the ligand in those rats was significantly lower than that in the MIA_PPI-low_ group.

As in the case of MIA, the acute injection of Poly I:C in adulthood affected only the MIA_PPI-high_ offspring (Table 3). The mRNA level of *Cx3cl1* was reduced in the hippocampi of the MIA_PPI-high_ rats compared to the control and the MIA_PPI-low_ animals after the additional stimulation with Poly I:C. Additionally, the hippocampal expression of *Cx3cr1* was lower in the MIA_PPI-high_ group subjected to the “second hit” with Poly I:C than in the control offspring (Table 3).

We did not observe any dysfunctions of the *Cd200*-*Cd200r* axis after MIA or acute treatment with Poly I:C in the frontal cortices or hippocampi of the examined groups.

### 2.8. Levels of CX3CL1, CX3CR1, CD200, and CD200R Proteins in the Frontal Cortices and Hippocampi of Adult Male Offspring

**Figure 7:**
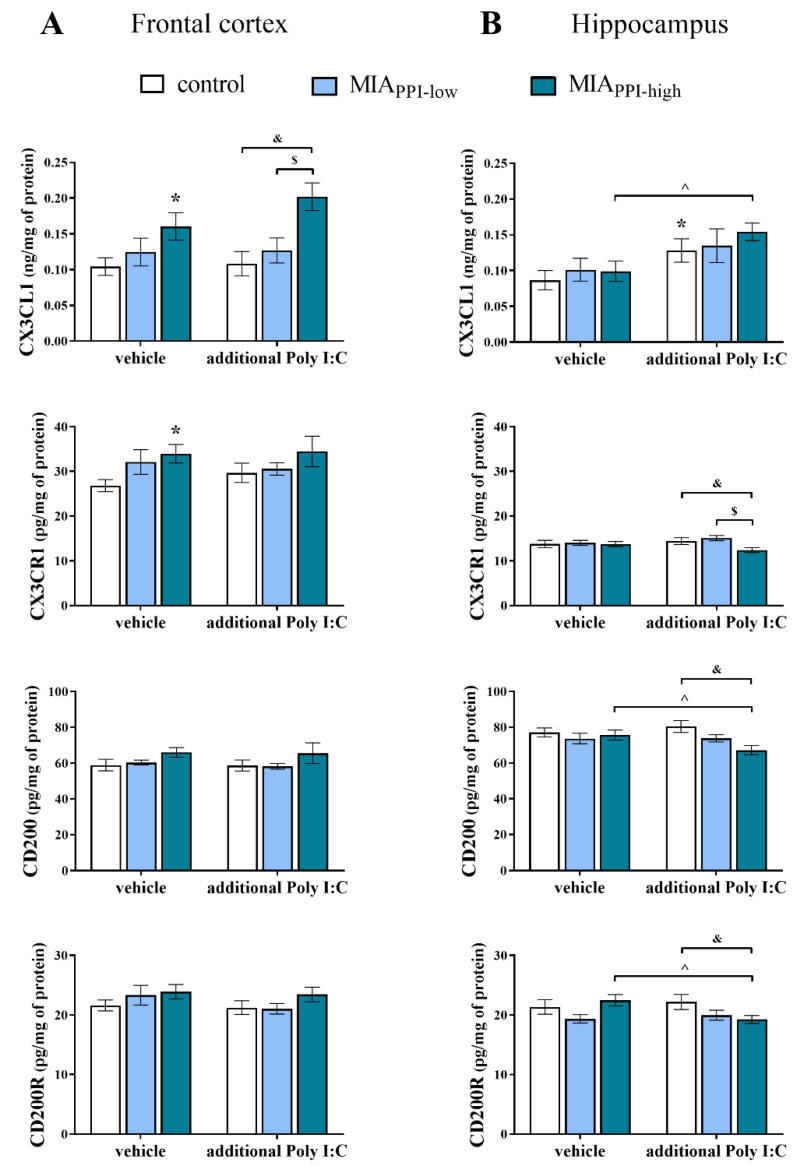
Impact of maternal immune activation (MIA) and the additional acute challenge with polyinosinic:polycytidylic acid (Poly I:C) on the protein levels of CX3CL1, CX3CR1, CD200, and CD200R in the frontal cortices. (**A**) and hippocampi (**B**) of male Sprague-Dawley offspring at postnatal day 120 (PND120). *n* = 6–9 in each group. The results are presented as the means ± standard errors of the mean (SEM). * *p* < 0.05 vs. control + vehicle, ^ *p* < 0.05 vs. MIA_PPI-high_ + vehicle, $ *p* < 0.05 vs. MIA_PPI-low_ + Poly I:C, & *p* < 0.05 vs. control + Poly I:C.

In the next step of the study, we determined the protein levels of the systems controlling neuron-microglia interactions in the brains of adult rats after MIA and the additional acute immune stimulation with Poly I:C (Figure 7). In agreement with the mRNA results, changes in the CX3CL1-CX3CR1 network were mainly present in the MIA_PPI-high_ animals. More precisely, in the frontal cortex, MIA increased the CX3CL1 and CX3CR1 levels. The results revealed that after the additional injection of Poly I:C, the cortical level of CX3CL1 in the MIA_PPI-high_ offspring was higher than that in the MIA_PPI-low_ and control groups. In the frontal cortex, the CD200-CD200R pathway was affected neither by MIA nor by the “second hit” with Poly I:C (Figure 7A).

In the hippocampus, we did not observe the influence of MIA on the examined ligand-receptor axes (Figure 7B). The alterations were revealed only after acute Poly I:C treatment in the form of increased CX3CL1 and reduced CD200 and CD200R levels in the hippocampi of the MIA_PPI-high_ animals. The hippocampal levels of CX3CR1, CD200, and CD200R in the MIA_PPI-high_ rats subjected to Poly I:C in adulthood were significantly lower than that in the control offspring, while the CX3CR1 level was also reduced compared to the MIA_PPI-low_ animals (Figure 7B).

### 2.9. IBA1 Levels in the Frontal Cortices and Hippocampi of Adult Male Offspring

**Figure 8:**
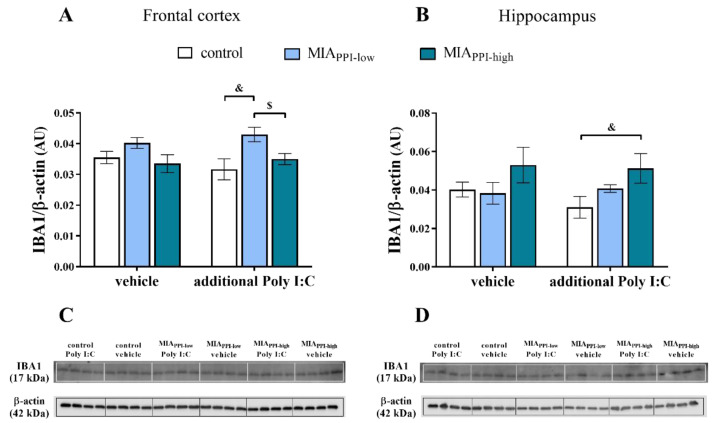
Impact of maternal immune activation (MIA) and the additional acute challenge with polyinosinic:polycytidylic acid (Poly I:C) on the protein level of IBA1 in the frontal cortices (**A**) and hippocampi (**B**) of male Sprague-Dawley offspring at postnatal day 120 (PND120). *n* = 3–4 in each group. The results are presented as the IBA1/β-actin ratio ± standard errors of the mean (SEM). $ *p* < 0.05 vs. MIA_PPI-low_ + Poly I:C, & *p* < 0.05 vs. control + Poly I:C. (**C**,**D**) Representative immunoblots for each group.

Next, we determined IBA1 levels in the homogenates of the frontal cortices and hippocampi of the animals after MIA and the additional challenge with Poly I:C (Figure 8). Western blot analysis revealed no significant effect of MIA on the levels of IBA1 in the studied brain areas of the offspring from any examined group.

The “second hit” resulted in an elevated cortical level of IBA1 in the MIA_PPI-low_ rats compared to the control animals that were also treated with Poly I:C in adulthood (Figure 8A). The level of IBA1 in the frontal cortices of the MIA_PPI-high_ offspring exposed to stimulation with Poly I:C was lower than that in the MIA_PPI-low_ group.

Considering the hippocampus, the only difference observed was an increase in the IBA1 level detected for the MIA_PPI-high_ rats after the additional injection of Poly I:C when compared to the control offspring challenged with the “second hit” (Figure 8B).

## 3. Discussion

The most important finding presented in our study is that MIA produced by Poly I:C treatment in the late period of gestation leads not only to behavioural alterations in adult offspring but also to changes in neuron-microglia proteins in certain areas of the brain in adulthood. The disturbances, mainly in the CX3CL1-CX3CR1 dyad, can imply a shift in the reactivity of microglial cells and/or can modulate the inflammatory response after the “second hit”, and these changes may in part underlie behavioural disturbances in adult offspring.

Poly I:C is a commercially available synthetic analogue of double-stranded RNA (dsRNA). dsRNA is generated during viral infection as a replication intermediate for single-stranded RNA (ssRNA) or as a byproduct of symmetrical transcription in DNA viruses [67,68]. Generally, recognition by the immune system and binding to the transmembrane protein Toll-like receptor 3 (TLR3) stimulates the production of various proinflammatory factors (such as cytokines and chemokines). Although the Poly I:C is widely used in preclinical research to create MIA models, it should be stressed that the immunological changes it induces in rodents are time-dependent, limited in time [5], and dependent on the precise dosage [69,70,71]. In the present study, we used a single intravenous injection of Poly I:C to pregnant females on gestational day 15 (GD15) and observed that in such a regimen, administration of Poly I:C leads to both negative and positive symptoms in adult rat offspring, although expressed with varying potency.

Among the negative symptoms, anxiety and social withdrawal are frequently noticed in patients with schizophrenia [56,72]. In the present study, we did not observe the effect of MIA on anxiety behaviour assessed in the light-dark box test. This finding seems to be consistent with the data of Vorhees et al. [13], who described no impact of Poly I:C treatment on that parameter. It should be highlighted, however, that in their research this immune stimulant was administered several times (GD14–18). In contrast, experiments carried out in the same experimental paradigm in Wistar rats revealed that Poly I:C triggered an anxiolytic phenotype, expressed as reduced fear of open spaces in adult offspring and the elevation of psychomotor activity [73]. Therefore, it seems that not only the experimental regimen but also the genetic background is important in the expression of changes in adulthood after Poly I:C. Those aspects may also explain the variation in the performance in the social interaction test. We demonstrated the influence of MIA on the social behaviour of adult male Sprague-Dawley offspring, which was expressed as a diminished number of events and reduced time spent on aggressive behaviour. Several articles indicate a similar effect of Poly I:C-elicited MIA on that characteristic. Among them, the results obtained in Wistar offspring rats conducted in the same experimental setting showed that social interactions were shifted towards enhanced aggressive behaviour [73]. Also, in animal models wherein Poly I:C was administered to pregnant mice, an increase in the presence of an aggressive phenotype in adult offspring was highlighted [74,75,76].

Negative symptoms of schizophrenia are sometimes combined with the depressive phenotype [77,78]. Therefore, we assessed the MIA effect in the FST, which is a method used to estimate depressive-like behaviour in experimental animals [43,79,80]. As demonstrated in the present study, MIA increased the immobility time and thus decreased swimming and climbing behaviour in the Porsolt test. Although the behavioural parameters we evaluated in the present study are governed by numerous mechanisms and are mediated by various areas of the brain, the mutual interaction between them gives a fairly homogeneous phenotype. Overall, even though no changes in the light-dark box test were observed in adult male offspring, we found less aggressive interactions, which may suggest a fear of contact with other animals and thus may explain the reduced number of aggressive episodes that are often seen in the resident-intruder test. Moreover, the increase in depressive-like changes suggests withdrawal and indicates a schizoaffective phenotype of adult offspring after MIA in the present research.

Since time-window studies in the Poly I:C model postulate that the distinction between early/late pregnancy immune challenge may correspond to the positive-negative dichotomy in schizophrenia [81,82], we also assessed the potential impact of MIA generated on GD15 on some schizophrenia-like positive symptoms. We demonstrated an increase in exploratory activity related to the collection of information about unfamiliar parts of the environment, which confirmed the high levels of motivation to explore in Sprague-Dawley rats [60]. Thus, the negative correlation between novelty-related behaviour, immobility, and climbing time in the FST, which has been postulated by some authors [83,84], has been confirmed. Our present results are consistent with previously published data from the MIA model induced by the administration of the bacterial endotoxin lipopolysaccharide (LPS) [85] as well as with the results of other authors obtained in neurodevelopmental models of schizophrenia [86]. These observations positively validate the Poly I:C MIA model in the context of the presence of positive symptoms in animals, which can be compared with the psychomotor agitation present in some schizophrenic patients [87].

The PPI is a cross-species phenomenon [88], thereby providing a very useful paradigm for translational schizophrenia research [89,90,91]. PPI is an operational measure of the preattentive filtering process known as sensorimotor gating, which reflects the neural filtering of redundant or unnecessary stimuli that takes place in complex systems [89,91,92]. Our study showed age-dependent alterations in the PPI evoked by Poly I:C in Sprague-Dawley offspring. More specifically, the changes were not present in the offspring on PND30 and PND60 but appeared in adulthood on PND100. Interestingly, the analysis revealed a dichotomy in the profile of results from the PPI test, namely, both reduced (MIA_PPI-low_) and increased (MIA_PPI-high_) PPI was demonstrated when compared to the control animals. Our present data contradict previously reported findings obtained in Wistar rats, where in the same experimental model, PPI deficits induced by Poly I:C treatment were detected in the greater part of adult offspring [73]. The phenomenon observed herein is difficult to explain and determine whether both displayed phenotypes (MIA_PPI-low_ and MIA_PPI-high_) should be considered behavioural deficits or malfunction. In the literature, the MIA-generated changes in PPI differed between experiments, although most of them showed deficits [9,93,94] or lack of changes [95,96]. However, in accordance with our observations, Vorhees et al. [13] noted an increased PPI% in the Poly I:C group. Certainly, the discrepancies between the results are influenced by many factors, including genetics, environmental aspects, and the time window of prenatal treatment with Poly I:C. These factors strongly modulate the developmental vulnerability to postnatal brain dysfunctions. Moreover, these factors may also lead to divergent presence or masking the time-dependent behavioural and cognitive alterations in adult offspring induced by MIA. Based on this observation and in line with the “two-hit” hypothesis of schizophrenia, we introduced an additional stimulus in the form of acute systemic Poly I:C treatment on PND120. Unexpectedly, we did not detect the impact of this challenge in any investigated group. To the best of our knowledge, this is the first such report in Sprague-Dawley rats, which may suggest the lack of behavioural response to the second stimulus in adult life in offspring prenatally exposed to Poly I:C in the late period of gestation.

Many data postulate that Poly I:C-evoked MIA profoundly affects offspring behaviour depending on the profile of immune alterations [95,97,98,99]. Nonetheless, the mechanism of this phenomenon has not yet been precisely defined. Although it has been suggested that changes in the activity of immunocompetent cells in the brain, including microglia, contribute to the appearance and course of the behavioural profile, these data are still controversial. Therefore, the second main purpose of the present study was to characterize the potential influence of neuron-microglia proteins, which are endogenous axes regulating immune homeostasis in the brain, on the microglial reactivity profile in the frontal cortices and hippocampi of adult offspring prenatally exposed to MIA. Remarkably, the potential role of the “second hit” in the modulation of microglial trajectory in all tested groups (control, MIA_PPI-low_, and MIA_PPI-high_) in the PPI test was scrutinized.

Unexpectedly, we did not find an impact of MIA on the CD200-CD200R ligand-receptor axis in the model we used.

The most striking findings from our research reveal the changes in the expression of CX3CL1-CX3CR1 neuron-microglia proteins in the examined brain areas; however, these observations were noticeable only in the offspring without deficient PPI (MIA_PPI-high_). Specifically, in the frontal cortex of those animals, we found enhanced CX3CL1-CX3CR1 protein levels, which might indicate an augmented synaptic reconstruction occurring after Poly I:C in some offspring [100]. In the hippocampus, we observed down-regulation of *Cx3cl1*-*Cx3cr1* only in the offspring without PPI reduction (MIA_PPI-high_). This finding may suggest that MIA triggers changes not only in transcriptional regulation but also in translational processes and translocation [101]. This phenomenon may, at least in part, explain the opposite results, concerning the levels and mRNA expression of these proteins, observed in our study. In the brain, CX3CL1 signalling has been demonstrated to facilitate the migration and cell adhesion of CX3CR1-expressing microglia to neuronal sites [102]. Furthermore, the CX3CL1-CX3CR1 dyad contributes to the regulation of synaptic organization [103,104], hippocampal cognitive function, and neurogenesis [105,106] as well as to a reduction in excitotoxicity [107]. Since the functional basis of PPI is regulated by interactions between various structures, including hippocampal and cerebral inputs [108,109], as well as by dopamine [108,110] and serotonin transmission [111,112], and CX3CL1 participates in the regulation of these neurotransmitters, the changes in this signalling observed between the offspring groups (MIA_PPI-low_ and MIA_PPI-high_) may be reflected by differences in behavioural schizophrenia-like patterns. However, this hypothesis should be a focus of further studies.

Support for the role of the CX3CL1-CX3CR1 pathway in psychiatric disorders comes from a meta-analysis of microarray data demonstrating a significant decrease in *CX3CR1* expression in the postmortem brain and blood of individuals with schizophrenia [113]. Recently, Hill et al. [114] provided evidence for disrupted CX3CL1 levels in schizophrenic patients and speculated that disturbances in this chemokine signalling might contribute to neuron-microglia disconnectivity in this disorder. On the other hand, Zhang et al. [115] reported that there were no differences between *CX3CR1* expression in patients with schizophrenia and controls. Thus far, the preclinical data are inconclusive. For instance, in DBA/2 mice, schizophrenia-related behaviour (expressed as PPI deficit and reduced social interactions) coexists with the down-regulation of *Cx3cl1* gene expression in the cortex [116]. Moreover, Zhan et al. [117] indicated that *Cx3cr1*-/- mice showed alterations in social and repetitive behaviours, while Zhou et al. [118] demonstrated the lack of changes in the PPI in *Cx3cr1*-/- transgenic mice in a social isolation model of schizophrenia. Thus, it is not possible to unequivocally resolve whether the increase in CX3CL1-CX3CR1 levels in the frontal cortex observed in our study can be regarded as a factor able to prevent a deficit in the PPI test. Nevertheless, in the offspring without a reduction in PPI (MIA_PPI-high_), we showed an increase in *Igf-1* expression in the frontal cortex and the down-regulation of *MhcII* in the hippocampus. Altered serum levels of IGF-1 have also been found in schizophrenic patients and are associated with psychopathological symptoms [119]. IGF-1 expression was correlated with cognitive performance in the individuals suffering from schizophrenia [119], indicating that the enhanced *Igf-1* expression we observed might be protective against schizophrenia-associated cognitive impairments. Notably, several authors have recently pointed out that increased IGF-1 levels may also counter depressive symptoms [120,121,122]. In light of the involvement of MHCII in the inflammatory response, the processes related to neurodevelopment and behaviour [123] and the down-regulation of *MhcII* expression in the hippocampus observed only in the offspring without reduced PPI (MIA_PPI-high_) may be responsible for modification of the MIA-generated inflammatory response. Therefore, it can be speculated that this change, at least in part, prevents the occurrence of the deficit in the group of male offspring (as demonstrated in the MIA_PPI-high_ group) or modifies the susceptibility of specific areas of the brain to the “second hit”. We observed that the additional stimulus in adulthood (as systemic Poly I:C injection) reduced the expression of *Cx3cl1-Cx3cr1* and led to a decrease in the CD200-CD200R levels, mainly in the hippocampus of the offspring without a PPI deficit (MIA_PPI-high_). Since CX3CL1-CX3CR1 and CD200-CD200R dyads play a role as a feedback mechanism for limiting microglial activation [41,124,125], in the next part of our research, we analysed the potential impact of the above-described changes in ligand-receptor axes on microglial trajectory influencing, in particular, behavioural differentiation in response to the PPI test. Although we did not demonstrate the impact of MIA on the proinflammatory phenotype of microglia of adult Sprague-Dawley offspring, the “second hit” resulted in the up-regulation of *Cd40*, *iNos*, *Il-1β*, *Tnf-α,* and *Il-6* expression in the hippocampus. Simultaneously, we observed a lack of differences in the proinflammatory status of microglia between the offspring groups distinguished above (MIA_PPI-low_ and MIA_PPI-high_). Thus, it follows that deficits in the hippocampal neuron-microglia axes induced in our model by the “second hit” are not secondary to the microglial response; however, this hypothesis warrants further examination. This finding is partially consistent with other models showing no evidence for overt microglial abnormalities between control and MIA-treated offspring [126]. Furthermore, increased microglial density with characteristics of nonreactive activation associated with behavioural schizophrenia-like disturbances has been shown by using Poly I:C-induced MIA [96]. Some data indicate that schizophrenia-associated PPI deficits develop as a result of adolescent microglia activation, which is transient and not visible in the Poly I:C-evoked MIA model in adulthood [100], as presented in our research. Unfortunately, to the best of our knowledge, there are no data about the “two-hit” hypothesis of schizophrenia in the regimen of Poly I:C treatment we presented within this article.

Another finding of crucial significance from our study is the observation that the “second hit” triggers the increase in the expression of *Cd40* and *iNos* and leads to an exaggerated proinflammatory response, which is reflected as *Il-1β*, *Tnf-α,* and *Il-6* gene up-regulation in the frontal cortex of MIA offspring. The “priming” potential of microglial reactivity in schizophrenia-sensitive areas of the brain may directly lead to cognitive and negative symptoms and an array of malfunctions associated with positive symptoms of the condition, which we observed only in the offspring without a deficit in PPI (MIA_PPI-high_). This phenomenon is difficult to explain and further complicated by the lack of behavioural changes observed in animals in response to the “second hit”. On the other hand, the reduced *Cx3cr1* expression after the additional injection of Poly I:C may drive the neuroinflammatory priming of microglia, while the cortical loss of control may explain the disinhibition of subcortical dopamine signalling causing psychotic symptoms [127]. Proinflammatory cytokines play an important role in the mediation of these changes. For instance, IL-1β up-regulation potentiates neurodegeneration induced by dopaminergic signalling [49], whereas excessive secretion of TNF-α contributes to cognitive impairment [128]. Nonetheless, the role of IL-6 increase seems to be pivotal not only in the induction of behavioural disturbances in the model based on the Poly I:C-produced MIA challenge but also in the priming of microglia. In adult neurons, elevated IL-6 levels are related to dysfunction of GABAergic parvalbumin-containing inhibitory neurons [129]. In our previous study, we demonstrated that elevated IL-6 levels preceded the manifestation of schizophrenia-like disturbances [130]. Additionally, acute IL-6 treatment was shown to trigger a deficit in sensorimotor gating in mice [19], while in Sprague-Dawley rats in the LPS-induced MIA model, elevated *Il-6* expression participated in the manifestation of the PPI deficit, negative regulation of the CD200-CD200R axis, and microglia priming [85]. Based on the mentioned data, the time of the tests performed after applying the “second hit” could have influenced the rate of transcription and translation processes as well as the insufficient increase in the level of *Il-6* and the lack of expected behavioural effects in offspring with “primed microglia”.

## 4. Materials and Methods

### 4.1. Animals

Sprague-Dawley rats were purchased from Charles River (Sulzfeld, Germany) and maintained under standard conditions: room temperature of 23 °C, light/dark cycle of 12/12 h, lights on at 6:00 am and ad libitum access to water and food. During the proestrus phase, female rats were placed with males for 12 h and the presence of sperm in vaginal smears was checked the next morning. The pregnant females were then randomly assigned to two equal groups: (1) control and (2) MIA (*n* = 10 in each group). All procedures were approved by the Animal Care Committee of the Maj Institute of Pharmacology, Polish Academy of Sciences, Cracow, and met the criteria of the International Council for Laboratory Animals and Guide for the Care and Use of Laboratory Animals (consent number 128/2018 was issued exclusively on experiments using males; the females were included in another study not presented within this article). All possible efforts were made to minimise the number of animals used and their suffering.

### 4.2. Drugs and Treatment

#### 4.2.1. Prenatal Administration of Poly I:C

**Figure 9:**
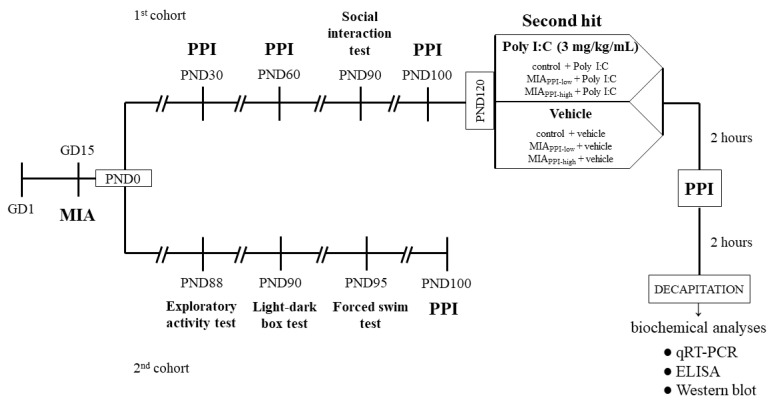
Schedule of the experimental design. Pregnant rats were exposed to maternal immune activation (MIA) with polyinosinic:polycytidylic acid (Poly I:C) (4 mg/kg in 1 mL, intravenously) on gestational day 15 (GD15). Control animals were subjected to vehicle (saline) injections in a corresponding manner. Twenty-one days after birth (PND21), male offspring were separated from dams and housed in groups of 5 per cage under standard conditions. Before further experiments, the rats were divided into 2 cohorts. The offspring from the 1st cohort (both the control and the MIA groups) underwent the behavioural examination in the following order: the prepulse inhibition (PPI) test at PND30 and PND60, the social interaction test at PND90, and the PPI test at PND100. At PND120, the animals were divided into 6 groups (control + vehicle, control + Poly I:C, MIA_PPI-low_ + vehicle, MIA_PPI-low_ + Poly I:C, MIA_PPI-high_ + vehicle, MIA_PPI-high_ + Poly I:C) and were exposed to the “second hit”, according to the group assigned, either with Poly I:C (3 mg/kg in 1 mL, intraperitoneally) or vehicle (saline). Two hours later, the rats underwent the PPI test, and after another 2 h, they were sacrificed by decapitation. The tissues (the frontal cortices and hippocampi) were collected for biochemical analyses (qRT-PCR, ELISA, and Western blot). Simultaneously, the offspring from the 2nd cohort (both the control and the MIA groups) were subjected to behavioural examination, including exploratory activity test at PND88, light-dark box test at PND90, forced swim test (FST, Porsolt test) at PND95, and PPI test at PND100. The animals from the 2nd cohort were not included in the biochemical analyses.

Poly I:C was purchased from Sigma-Aldrich (St. Louis, MO, USA) as a sodium salt and dissolved in saline to obtain 1 mL of a 4 mg/kg solution. Poly I:C was administered to the tail vein of pregnant rats in the MIA group on GD15, while the control group received a corresponding injection of saline (vehicle) [16,94]. Male offspring were separated from dams twenty-one days after birth and kept in groups of five per cage under standard conditions for further experiments. Next, the rats were divided into two cohorts: the 1^st^ was used for the behavioural examinations and biochemical analyses, while the 2^nd^ simultaneously underwent behavioural tests. The behavioural experiments were performed between 9:00 a.m and 12:00 a.m. An overview of the experimental design is illustrated in Figure 9. The investigators were not blinded to the experimental conditions. The numbers of animals included in each analysis are presented in the description of the corresponding figure or table.

#### 4.2.2. Additional Immune Activation with Poly I:C in Adulthood

The solution of Poly I:C (Sigma-Aldrich, St. Louis, MO, USA) in saline at a concentration of 3 mg/kg in 1 mL was administered intraperitoneally [131] to male offspring from the control + Poly I:C, MIA_PPI-low_ + Poly I:C and MIA_PPI-high_ + Poly I:C groups at PND120. The control + vehicle, MIA_PPI-low_ + vehicle, and MIA_PPI-high_ + vehicle groups received an appropriate injection of vehicle (saline).

### 4.3. Behavioural Tests

#### 4.3.1. Light-Dark Box Test

The light dark-box test was performed according to the procedure described by Chocyk et al. [132]. An apparatus consisting of four cages with a computer-controlled system (TSE Systems, Bad Homburg, Germany) was used for the test. Each experimental box had two compartments: light (covering ¾ of the cage, brightly lit—100 lx) and dark (covered with a lid), made of transparent and black acrylic, respectively. Both chambers were permeable to infrared light and were connected by a central gate (10.6 cm × 10.4 cm). These two parts of the cage were freely available to animals for exploration. The experimental boxes were placed in soundproof, ventilated cabinets on the basis of integrated infrared sensors along the horizontal and vertical axes. The male rats at PND90 from the control and MIA groups were kept in darkness for an hour before the test. The whole experiment was carried out in a dark room. At the beginning of each 10-min test session, the animal was placed in one corner of the light compartment, facing away from the gate. The behavioural response of rats during the trials was recorded by Fear Conditioning Software (TSE, Bad Homburg, Germany). In particular, the time spent in each compartment, the distance travelled, and the average speed were calculated for each animal.

#### 4.3.2. Social Interaction Test

The social interaction tests were conducted based on the resident-intruder paradigm [133] using the procedure described previously by Wedzony et al. [86]. Male offspring rats (the residents) at PND90 were housed in pairs (four pairs of control and four pairs of MIA animals) in standard laboratory cages. Other naïve male rats of a similar age were used as intruders. The residents and intruders had never been in contact before the experiments. Three hours prior to the test, one of the two residents was removed from the home cage, which was placed in the arena with constant illumination. The intruder animal was positioned in the resident’s home cage, and the time and number of the resident’s behavioural responses were recorded during a 15-min session. The following activities were assessed: (1) non-aggressive, containing sniffing (sniffing the body parts of an intruder rat, including anogenital region) and social grooming (licking and chewing a fur of an intruder), and (2) aggressive, involving attack, fight, and aggressive grooming (aggressive licking and chewing a fur of an intruder rat). Each intruder animal was used only once, and the experiments for other residents of the same cage were performed the following week. Social interactions are presented as summed scores of the time and the number of aggressive and non-aggressive activities.

#### 4.3.3. Forced Swim Test

The FST was conducted according to a previously described method [134] commonly used in our laboratory [80,135,136,137,138]. The male offspring at PND95 were individually subjected to two trials during which they were forced to swim in a cylinder (50 cm high, 18 cm in diameter) filled with water (23 °C) to a height of 35 cm. There was a 24-h interval between the trials. The first trial lasted 15 min, while the second trial continued for 5 min. The total times of immobility, mobility (swimming) and climbing were measured by the observer throughout the second trial.

#### 4.3.4. Exploratory Activity Test

The exploratory activity of the control and MIA rats was performed at PND88 according to the procedure previously described by Basta-Kaim et al. [139]. The exploration was recorded individually for each animal in Opto-Varimex cages (Columbus Instruments, Columbus, OH, USA) connected to an IBM-PC compatible computer. Each cage (43 cm × 44 cm) was provided with 15 infrared emitters on the horizontal and vertical axes and an equivalent number of receivers on opposite walls. Exploratory activity was determined based on a crossing of three consecutive photobeams by an animal, and it was presented both as the distance travelled in respective time intervals (5 min each) and as the total distance during 30-minute-interval.

#### 4.3.5. Prepulse Inhibition Test

PPI was performed based on our previously published studies [66,130,140] with some modifications. The procedure was carried out on the male offspring at four time points: at PND30, PND60, PND100, and at PND120—2 h after the additional injection of Poly I:C. PPI was performed in eight ventilated startle chambers (SR-LAB, San Diego Instruments, California, USA) with a single Plexiglas cylinder (inner diameter of 9 cm) mounted in each of them. A high-frequency loudspeaker inside each chamber produced a continuous background noise of 65 dB and various acoustic stimuli. A piezoelectric accelerometer was used to determine the average startle amplitudes (AVGs) for each animal, which were then digitized and used in subsequent analyses. Before placing the animals in the chambers, each of them was individually calibrated by an external sensor to display a similar reference stimulus reading. The AVGs were measured in the registration window of 200 ms. After 5 min of habituation with background noise, the animals were randomly subjected to four types of acoustic stimuli. Each experimental trial consisted of either a single pulse [intensity: 120 dB, duration: 40 ms, (P)] or a pulse preceded by a prepulse at one out of three intensities [70, 75, 80 dB; duration: 20 ms; (PP)] applied 80 ms before the pulse. In each session, 20 trials of each type were presented with an interstimulus interval of 20 s. The AVG values were recorded, and the percentage of PPI (PPI%) induced by each prepulse intensity was calculated as PPI% = [(P − PP)/P] × 100%.

At PND100, the offspring from the MIA group were divided into two categories: MIA_PPI-low_ (with the deficit in PPI) and MIA_PPI-high_ (without the deficit). The subcategorization was performed based on the PPI results calculated with the AVGs for the 75 dB prepulse. First, the mean response in PPI at 75 dB prepulse was calculated for the control group. Then, the MIA offspring were divided in such a way that all animals with PPI% lower than the average response of the control rats were categorized as “MIA_PPI-low_”, and all animals with PPI% higher than the mean for the control group were assigned to the “MIA_PPI-high_” group. Categories obtained for 75 dB prepulse were maintained for the remaining prepulse intensities (70 and 80 dB).

### 4.4. Biochemical Analyses

#### 4.4.1. Tissues Collection and Preparation

The frontal cortices and hippocampi were collected from adult animals 4 h after the additional injection of Poly I:C or saline. The tissues were dissected on an ice-cold glass plate and stored at −80 °C for further testing. The frontal cortices and hippocampi were homogenized with RIPA lysis buffer containing protease inhibitor cocktail, phosphatase inhibitor cocktail, 1 mM sodium orthovanadate, and 1 mM phenylmethanesulfonyl fluoride (all from Sigma-Aldrich, St. Louis, MO, USA) by Tissue Lyser II (Qiagen Inc., Valencia, CA, USA). A BCA Protein Assay Kit (Sigma-Aldrich, St. Louis, MO, USA) was used to determine the protein concentration in the test samples according to the manufacturer’s instructions with bovine serum albumin (BSA) as a standard and was measured at 562 nm in a Tecan Infinite 200 Pro spectrophotometer (Tecan, Mannedorf, Germany). Collected samples were then used for biochemical analysis by applying enzyme-linked immunosorbent assay (ELISA) and Western blot techniques.

#### 4.4.2. Quantitative Real-Time Polymerase Chain Reaction

Total RNA was isolated from the frontal cortices and hippocampi of male rats at PND120 using the GeneMATRIX Universal RNA Purification Kit (EURx, Gdańsk, Poland) strictly according to the manufacturer’s instructions. The samples were homogenized in lysis buffer supplied with a kit by Tissue Lyser II (Qiagen Inc., Valencia, CA, USA). The RNA concentration was determined using a NanoDrop ND-1000 Spectrometer (Thermo Fisher, Waltham, MA, USA). Equal amounts of RNA (1 μg) were reverse transcribed into complementary DNA (cDNA) using an NG dART RT kit (EURx, Gdańsk, Poland). The cDNA was amplified with FastStart Universal Probe Master (Rox) kit (Roche, Basel, Switzerland), TaqMan probes and primers for the genes: *Cx3cl1* (Rn00593186_m1), *Cx3cr1* (Rn00591798_m1), *Cd200* (Rn01646320_m1), *Cd200r* (Rn00576646_m1), *MhcII* (Rn01424725_m1), *Cd40* (Rn01423583_m1), *iNos* (Rn00561646_m1), *Il-1β* (Rn00580432_m1), *Tnf-α* (Rn00562055_m1), *Il-6* (Rn01410330_m1), *Arg1* (Rn00691090_m1), *Igf-1* (Rn00710306_m1), *Tgf-β* (Rn00572010_m1), *Il-4* (Rn01456866_m1), and, as the reference, *Hprt* (Rn01527840_m1) or *B2m* (Rn00560865_m1) (all obtained from Life Technologies, Carlsbad, CA, USA). Amplification was performed in a 20-μL mixture consisting of cDNA, which was used as the polymerase chain reaction (PCR) template (1 μL), 1× FastStart Universal Probe Master (Rox) mix containing 250 nM hydrolysis probe labelled with the fluorescent reporter dye [fluorescein (FAM)] at the 5′-end and a quenching dye at the 3′-end (10 μL), and, finally, the remainder of PCR-grade distilled water (8 μL). The thermal cycling conditions contained an initial denaturation at 95 °C for 10 min, followed by 45 cycles of denaturation at 95 °C for 15 s, annealing at 60 °C for 1 min, and extension at 50 °C for 2 min. The threshold value (C_t_) for each sample was set in the exponential phase of PCR, and the ∆∆C_t_ method was used for the data analysis.

#### 4.4.3. Enzyme-Linked Immunosorbent Assay

The concentrations of CX3CL1 (Cloud-Clone Corp., Katy, TX, USA), CX3CR1, CD200, and CD200R (all from Cusabio, Houston, TX, USA) in the frontal cortices and hippocampi of male rats at PND120 were measured using commercially available ELISA kits according to the manufacturer’s instructions. The detection limits were as follows: CX3CL1: 0.055 ng/mL, CX3CR1: 5.8 pg/mL, CD200: 11.75 pg/mL, and CD200R: 4.67 pg/mL, and the intra- and interassay precision values were CX3CL1: <10%, <12%, CX3CR1, CD200, and CD200R: <8%, <10%.

#### 4.4.4. Western Blot

The samples containing 10 μg of protein were mixed with Laemmli sample buffer (Bio-Rad, Hercules, CA, USA) (4:1 ratio, *v*/*v*) and heated at 95 °C for 8 min in Eppendorf Thermomixer comfort (Sigma-Aldrich, St. Louis, MO, USA). Afterwards, all samples were resolved on 4–20% precast polyacrylamide gels (Bio-Rad, Hercules, CA, USA) under constant voltage (200 V) and transferred to PVDF membranes (Sigma-Aldrich, St. Louis, MO, USA) using Trans-Blot Turbo (Bio-Rad, Hercules, CA, USA). The blots were cut into two parts, rinsed 3× for 10 min with tris-buffered saline solution (TBS), and blocked in 5% BSA dissolved in TBS with 0.1% Tween 20 (TBST) (both from Sigma-Aldrich, St. Louis, MO, USA) for 1 h at room temperature (RT). After three 10-minute-long washes in TBST, the membranes were incubated overnight at 4 °C with anti-IBA1 (NBP2–19019, 1:500, Novus Biologicals, Centennial, CO, USA) or anti-β-actin (A5441, 1:10,000, Sigma-Aldrich, St. Louis, MO, USA) and antibody diluted in a SignalBoost Immunoreaction Enhancer Kit (Millipore, Warsaw, Poland). Next, the blots were rinsed 3× for 10 min with TBST and incubated with the appropriate peroxidase-conjugated secondary antibody: goat anti-rabbit IgG (PI-1000, 1:2500) or horse anti-mouse IgG (PI-2000, 1:4000) (both from Vector Laboratories, Peterborough, UK) for 1 h at RT. Then, washing 3 times for 10 min in TBST was repeated. The immunocomplexes were detected using Pierce™ ECL Western Blotting Substrate (Thermo Fisher, Pierce Biotechnology, Carlsbad, CA, USA) and visualized using a Fujifilm LAS-1000 System (Fuji Film, Tokyo, Japan). The relative levels of immunoreactivity were densitometrically quantified with Fujifilm Multi Gauge software (Fuji Film, Tokyo, Japan).

### 4.5. Statistical Data Analysis

The results were analysed using Statistica 13.0 Software (StatSoft, Palo Alto, CA, USA). The data from behavioural studies are presented as the means ± standard errors of the mean (SEM). The results from qRT-PCR are displayed as the means of the average fold ± SEM, those from ELISA experiments are presented as the means ± SEM, and those from Western blot analyses are shown as the means of the IBA1/β-actin ratio ± SEM. Student’s t-test was performed to compare variables between groups in the light-dark box test, social interaction test, FST, exploratory activity, and PPI at PND30, PND60, and PND100. For the PPI after the additional injection of Poly I:C or saline at PND120 and all biochemical experiments (qRT-PCR, ELISA, Western blot), statistical analyses were performed with planned comparisons via a one-way ANOVA (contrast analysis). The results were considered statistically significant at *p* < 0.05. All precise data expressed as the means ± SEM and detailed statistics are provided in the Supplementary Materials. All graphs were prepared using GraphPad Prism 7 (San Diego, CA, USA).

## 5. Conclusions

In summary, an immune challenge in the last period of pregnancy in Sprague-Dawley rats leads to the appearance of schizophrenia-like disturbances in adult offspring. We demonstrated deficits expressed as a diminished number of aggressive interactions and the presence of depressive-like episodes, which may suggest withdrawal and indicate a schizoaffective phenotype of adult offspring after prenatal Poly I:C treatment. We observed an increase of exploratory activity and dichotomy in the sensorimotor gating in the PPI test as well as the lack of behavioural changes in the offspring after MIA to the second stimulus in adulthood. Moreover, our study is the first to raise the possibility that the changes in neuron-microglia proteins, mainly CX3CL1-CX3CR1, at least in part could be engaged in the regulation of behaviour by microglial trajectory in adult offspring rats. Although the presented data do not provide unambiguous insights into the MIA-induced alterations underlying differential behavioural status in the PPI test, they give important input indicating that the changes generated in the late period of gestation may go beyond the role of the altered CX3CL1-CX3CR1 axis and microglial trajectory in the development and manifestation of behavioural deficits in adulthood following the “two-hit” hypothesis of schizophrenia. Therefore, future efforts to identify the biological background of these interactions in the Poly I:C-induced MIA model in Sprague-Dawley rats are desirable to unequivocally clarify this issue.

## Figures and Tables

**Table 1:** Impact of maternal immune activation (MIA) on social (aggressive and non-aggressive) behaviour of adult male Sprague-Dawley offspring, measured in the test of social interactions. *n* = 8 in each group. The results are presented as the means ± standard errors of the mean (SEM). * *p* < 0.05 vs. control group.

Group	Type of Social Interaction			
	Aggressive		Non-Aggressive	
	Number of Events	Time (s)	Number of Events	Time (s)
control	4.50 ± 1.00	19.00 ± 5.01	18.88 ± 2.63	65.63 ± 12.64
MIA	0.50 ± 0.38 *	2.00 ± 1.36 *	18.75 ± 1.84	70.13 ± 10.78

**Table 2:** Impact of maternal immune activation (MIA) on prepulse inhibition (PPI) of the acoustic startle response in male Sprague-Dawley offspring at postnatal day 30 (PND30) and PND60. *n* = 15–18 in each group at PND30; *n* = 8 in each group at PND60. The results are presented as the means of the percentage of PPI (PPI%) induced by each prepulse intensity ± standard errors of the mean (SEM). Data were calculated on the basis of the average startle amplitudes (AVGs).

Prepulse Intensity	Group			
	PND30		PND60	
	Control	MIA	Control	MIA
70 dB	26.29 ± 5.30	28.71 ± 6.17	40.45 ± 4.50	40.98 ± 5.34
75 dB	44.43 ± 6.50	46.36 ± 5.77	63.29 ± 4.27	58.10 ± 6.07
80 dB	47.61 ± 5.56	46.05 ± 5.56	64.02 ± 4.11	61.52 ± 7.85

**Table 3:** Impact of maternal immune activation (MIA) and the additional acute challenge with polyinosinic:polycytidylic acid (Poly I:C) on the gene expression of *Cx3cl1*, *Cx3cr1*, *Cd200,* and *Cd200r* in the frontal cortices and hippocampi of male Sprague-Dawley offspring at postnatal day 120 (PND120). The mRNA levels were measured using qRT-PCR with *n* = 5–9 in each group. The results are presented as the average fold change ± standard errors of the mean (SEM). * *p* < 0.05 vs. control + vehicle, ^#^ *p* < 0.05 vs. MIA_PPI-low_ + vehicle, ^ *p* < 0.05 vs. MIA_PPI-high_ + vehicle, ^$^ *p* < 0.05 vs. MIA_PPI-low_ + Poly I:C, ^&^ *p* < 0.05 vs. control + Poly I:C.

**Factor**	**Gene Expression**					
	**Frontal Cortex**					
	**Control**		**MIA**			
			**PPI-Low**		**PPI-High**	
	**Vehicle**	**Poly I:C**	**Vehicle**	**Poly I:C**	**Vehicle**	**Poly I:C**
*Cx3cl1*	1.03 ± 0.09	1.11 ± 0.14	0.94 ± 0.13	0.74 ± 0.05 ^&^	0.78 ± 0.07	0.97 ± 0.10
*Cx3cr1*	1.05 ± 0.12	0.90 ± 0.07	1.01 ± 0.14	0.71 ± 0.09	1.01 ± 0.17	0.67 ± 0.06 ^
*Cd200*	1.01 ± 0.05	1.12 ± 0.13	1.03 ± 0.08	0.97 ± 0.11	0.94 ± 0.08	0.97 ± 0.09
*Cd200r*	1.04 ± 0.10	0.78 ± 0.18	0.83 ± 0.10	0.80 ± 0.11	0.89 ± 0.15	0.87 ± 0.15
**Factor**	**Hippocampus**					
	**Control**		**MIA**			
			**PPI-Low**		**PPI-High**	
	**Vehicle**	**Poly I:C**	**Vehicle**	**Poly I:C**	**Vehicle**	**Poly I:C**
*Cx3cl1*	1.03 ± 0.08	1.20 ± 0.08	0.94 ± 0.08	1.09 ± 0.12	0.70 ± 0.04 *^#^	0.82 ± 0.03 ^&$^
*Cx3cr1*	1.05 ± 0.13	1.02 ± 0.09	0.97 ± 0.09	0.89 ± 0.16	0.74 ± 0.04 *	0.76 ± 0.06 ^&^
*Cd200*	1.02 ± 0.07	1.09 ± 0.11	0.84 ± 0.08	0.95 ± 0.09	0.87 ± 0.07	1.04 ± 0.11
*Cd200r*	1.06 ± 0.12	1.40 ± 0.24	0.92 ± 0.07	1.34 ± 0.20	1.05 ± 0.10	0.93 ± 0.09

## Data Availability

All data supporting the conclusions of this manuscript are provided in the text, figures, tables, and Supplementary Materials.

## Supplementary Materials

The following are available online at https://www.mdpi.com/1422-0067/22/4/1558/s1.
