# Supplementary material for: Role of Polyinosinic:Polycytidylic Acid-Induced Maternal Immune Activation and Subsequent Immune Challenge in the Behaviour and Microglial Cell Trajectory in Adult Offspring: A Study of the Neurodevelopmental Model of Schizophrenia
Source: Int J Mol Sci. 2021 Feb 4;22(4):1558. doi: 10.3390/ijms22041558 (PMC7913889; doi:10.3390/ijms22041558)
Supplement: Supplementary file 1 [file ijms-22-01558-s001.pdf]

Supplementary materials

2. Results

2.1. Light-dark box test

No significant changes (Figure 1).

2.2. Social interaction test

– the time of aggressive behaviours: 19.00 ± 5.01 control vs. 2.00 ± 1.36 MIA, $F_{(1,14)}$ = 13.52, p = 0.0055 (Table 1)

– the number of aggressive behaviours: 4.50 ± 1.00 control vs. 0.50 ± 0.38 MIA, $F_{(1,14)}$ = 7.00, p = 0.0022 (Table 1)

2.3. Forced swim test

– the immobility time: 199.25 ± 9.41 control vs. 243.50 ± 13.88 MIA, $F_{(1,14)}$ = 2.18, p = 0.0195 (Figure 2)

– the swimming time: 100.75 ± 9.41 control vs. 56.50 ± 13.88 MIA, $F_{(1,14)}$ = 2.18, p = 0.0195 (Figure 2)

– the climbing time: 90.13 ± 9.81 control vs. 41.38 ± 8.77 MIA, $F_{(1,14)}$ = 1.25, p = 0.0024 (Figure 2)

2.4. Exploratory activity

– the total distance travelled: 2243.25 ± 125.70 control vs. 2772.94 ± 190.65 MIA, $F_{(1,25)}$ = 2.48, p = 0.0313 (Figure 3, inset)

– the exploration in the fourth interval of the experiment: 204.88 ± 27.90 control vs. 306.07 ± 34.12 MIA, $F_{(1,25)}$ = 1.61, p = 0.0317 (Figure 3)

2.5. Prepulse inhibition of the acoustic startle response

– PPI, the 70 dB prepulse, the animals at PND100: 46.19 ± 4.37 control vs. 64.73 ± 3.39 $MIA_{PPI\text{-}high}$, $F_{(1,37)}$ = 2.97, p = 0.0063 (Figure 4A)

– PPI, the 75 dB prepulse, the animals at PND100: 69.90 ± 2.71 control vs. 57.43 ± 2.55 $MIA_{PPI\text{-}low}$, $F_{(1,35)}$ = 2.35, p = 0.0065; 69.90 ± 2.71 control vs. 79.73 ± 2.30 $MIA_{PPI\text{-}high}$, $F_{(1,37)}$ = 2.47, p = 0.0193 (Figure 4A)

– PPI, the 80 dB prepulse, the animals at PND100: 70.12 ± 2.81 control vs. 55.60 ± 2.29 $MIA_{PPI\text{-}low}$, $F_{(1,35)}$ = 3.13, p = 0.0021; 70.12 ± 2.81 control vs. 79.49 ± 2.70 $MIA_{PPI\text{-}high}$, $F_{(1,37)}$ = 1.94, p = 0.0347 (Figure 4A)

– PPI, the 80 dB prepulse, the animals at PND120: 61.82 ± 6.44 $MIA_{PPI\text{-}low}$ + vehicle vs. 77.48 ± 4.84 $MIA_{PPI\text{-}high}$ + vehicle, $F_{(2,40)}$ = 3.20, p = 0.0335 (Figure 4B)

2.6. mRNA expression of microglial markers in the frontal cortices and hippocampi of adult male offspring

– Frontal cortex, *Cd40*: 1.00 ± 0.15 control + vehicle vs. 2.39 ± 0.47 control + Poly I:C, $F_{(5,31)}$ = 7.07, p = 0.0151; 1.08 ± 0.17 $MIA_{PPI\text{-}low}$ + vehicle vs. 3.38 ± 0.40 $MIA_{PPI\text{-}low}$ + Poly I:C, $F_{(5,31)}$ = 7.07, p = 0.0010; 0.88 ± 0.07 $MIA_{PPI\text{-}high}$ + vehicle vs. 3.26 ± 0.97 $MIA_{PPI\text{-}high}$ + Poly I:C, $F_{(5,31)}$ = 7.07, p = 0.0007 (Figure 5A)

- Frontal cortex, *iNos*: 1.01 ± 0.08 control + vehicle vs. 6.00 ± 1.47 control + Poly I:C, $F_{(5,24)}$ = 5.86, p = 0.0130; 0.53 ± 0.16 $MIA_{PPI\text{-}low}$ + vehicle vs. 7.45 ± 0.84 $MIA_{PPI\text{-}low}$ + Poly I:C, $F_{(5,24)}$ = 5.86, p = 0.0005; 1.14 ± 0.61 $MIA_{PPI\text{-}high}$ + vehicle vs. 6.21 ± 1.85 $MIA_{PPI\text{-}high}$ + Poly I:C, $F_{(5,24)}$ = 5.86, p = 0.0227 (Figure 5A)
- Frontal cortex, *Il-1β*: 0.95 ± 0.15 $MIA_{PPI\text{-}high}$ + vehicle vs. 2.74 ± 1.17 $MIA_{PPI\text{-}high}$ + Poly I:C, $F_{(5,32)}$ = 2.73, p = 0.0086; 1.20 ± 0.25 control + Poly I:C vs. 2.74 ± 1.17 $MIA_{PPI\text{-}high}$ + Poly I:C, $F_{(2,32)}$ = 3.26, p = 0.0177 (Figure 5A)
- Frontal cortex, *Tnf-α*: 0.84 ± 0.13 $MIA_{PPI\text{-}low}$ + vehicle vs. 3.90 ± 0.97 $MIA_{PPI\text{-}low}$ + Poly I:C, $F_{(5,32)}$ = 4.73, p = 0.0007; 0.99 ± 0.15 $MIA_{PPI\text{-}high}$ + vehicle vs. 2.87 ± 1.12 $MIA_{PPI\text{-}high}$ + Poly I:C, $F_{(5,32)}$ = 4.73, p = 0.0353; 2.13 ± 0.44 control + Poly I:C vs. 2.87 ± 1.12 $MIA_{PPI\text{-}high}$ + Poly I:C, $F_{(2,32)}$ = 2.56, p = 0.0309 (Figure 5A)
- Frontal cortex, *Il-6*: 0.98 ± 0.14 control + vehicle vs. 2.08 ± 0.16 control + Poly I:C, $F_{(5,31)}$ = 6.83, p = 0.0054; 1.29 ± 0.31 $MIA_{PPI\text{-}low}$ + vehicle vs. 2.23 ± 0.17 $MIA_{PPI\text{-}low}$ + Poly I:C, $F_{(5,31)}$ = 6.83, p = 0.0351; 1.27 ± 0.17 $MIA_{PPI\text{-}high}$ + vehicle vs. 3.00 ± 0.66 $MIA_{PPI\text{-}high}$ + Poly I:C, $F_{(5,31)}$ = 6.83, p = 0.0003; 2.08 ± 0.16 control + Poly I:C vs. 3.00 ± 0.66 $MIA_{PPI\text{-}high}$ + Poly I:C, $F_{(2,31)}$ = 2.68, p = 0.0331 (Figure 5A)
- Hippocampus, *MhcII*: 1.06 ± 0.11 control + vehicle vs. 0.71 ± 0.09 $MIA_{PPI\text{-}high}$ + vehicle, $F_{(5,36)}$ = 3.13, p = 0.0142 (Figure 5B)
- Hippocampus, *Cd40*: 1.02 ± 0.08 control + vehicle vs. 3.75 ± 0.82 control + Poly I:C, $F_{(5,37)}$ = 11.52, p = 0.0001; 0.91 ± 0.05 $MIA_{PPI\text{-}low}$ + vehicle vs. 5.10 ± 0.70 $MIA_{PPI\text{-}low}$ + Poly I:C, $F_{(5,37)}$ = 11.52, p = 0.0001; 0.84 ± 0.09 $MIA_{PPI\text{-}high}$ + vehicle vs. 3.74 ± 0.90 $MIA_{PPI\text{-}high}$ + Poly I:C, $F_{(5,37)}$ = 11.52, p = 0.0001 (Figure 5B)
- Hippocampus, *iNos*: 1.01 ± 0.28 control + vehicle vs. 6.42 ± 1.84 control + Poly I:C, $F_{(5,25)}$ = 6.24, p = 0.0005; 0.32 ± 0.06 $MIA_{PPI\text{-}low}$ + vehicle vs. 5.21 ± 0.95 $MIA_{PPI\text{-}low}$ + Poly I:C, $F_{(5,25)}$ = 6.24, p = 0.0047; 1.09 ± 0.48 $MIA_{PPI\text{-}high}$ + vehicle vs. 4.17 ± 0.73 $MIA_{PPI\text{-}high}$ + Poly I:C, $F_{(5,25)}$ = 6.24, p = 0.0478 (Figure 5B)
- Hippocampus, *Il-1β*: 1.07 ± 0.16 control + vehicle vs. 2.65 ± 0.80 control + Poly I:C, $F_{(5,37)}$ = 5.89, p = 0.0107; 1.08 ± 0.09 $MIA_{PPI\text{-}low}$ + vehicle vs. 3.07 ± 0.67 $MIA_{PPI\text{-}low}$ + Poly I:C, $F_{(5,37)}$ = 5.89, p = 0.0033; 0.82 ± 0.11 $MIA_{PPI\text{-}high}$ + vehicle vs. 2.38 ± 0.52 $MIA_{PPI\text{-}high}$ + Poly I:C, $F_{(5,37)}$ = 5.89, p = 0.0024 (Figure 5B)
- Hippocampus, *Tnf-α*: 1.05 ± 0.11 control + vehicle vs. 2.48 ± 0.44 control + Poly I:C, $F_{(5,37)}$ = 7.56, p = 0.0008; 1.35 ± 0.19 $MIA_{PPI\text{-}low}$ + vehicle vs. 3.69 ± 0.45 $MIA_{PPI\text{-}low}$ + Poly I:C, $F_{(5,37)}$ = 7.56, p = 0.0026; 0.86 ± 0.12 $MIA_{PPI\text{-}high}$ + vehicle vs. 2.84 ± 0.83 $MIA_{PPI\text{-}high}$ + Poly I:C, $F_{(5,37)}$ = 7.56, p = 0.0035 (Figure 5B)
- Hippocampus, *Il-6*: 1.05 ± 0.12 control + vehicle vs. 1.75 ± 0.23 control + Poly I:C, $F_{(5,37)}$ = 7.93, p = 0.0063; 0.73 ± 0.11 $MIA_{PPI\text{-}low}$ + vehicle vs. 2.79 ± 0.47 $MIA_{PPI\text{-}low}$ + Poly I:C, $F_{(5,37)}$ = 7.93, p = 0.0002; 0.81 ± 0.09 $MIA_{PPI\text{-}high}$ + vehicle vs. 1.98 ± 0.12 $MIA_{PPI\text{-}high}$ + Poly I:C, $F_{(5,37)}$ = 7.93, p = 0.0005 (Figure 5B)
- Frontal cortex, *Arg1*: 0.98 ± 0.11 control + Poly I:C vs. 0.73 ± 0.06 $MIA_{PPI\text{-}low}$ + Poly I:C, $F_{(2,34)}$ = 2.47, p = 0.0491 (Figure 6A)
- Frontal cortex, *Igf-1*: 1.02 ± 0.07 control + vehicle vs. 1.30 ± 0.14 $MIA_{PPI\text{-}high}$ + vehicle, $F_{(5,34)}$ = 3.47, p = 0.0319 ; 1.23 ± 0.12 $MIA_{PPI\text{-}low}$ + vehicle vs. 0.91 ± 0.07 $MIA_{PPI\text{-}low}$ + Poly I:C, $F_{(5,34)}$ = 3.47, p = 0.0245; 1.30 ± 0.14 $MIA_{PPI\text{-}high}$ + vehicle vs. 0.81 ± 0.06 $MIA_{PPI\text{-}high}$ + Poly I:C, $F_{(5,34)}$ = 3.47, p = 0.0018 (Figure 6A)
- Hippocampus, *Arg1*: 1.23 ± 0.13 $MIA_{PPI\text{-}low}$ + vehicle vs. 0.88 ± 0.06 $MIA_{PPI\text{-}high}$ + vehicle, $F_{(2,37)}$ = 2.84, p = 0.0232 (Figure 6B)

2.7. mRNA expression of Cx3cl1, Cx3cr1, Cd200 and Cd200r in the frontal cortices and hippocampi of adult male offspring

- Frontal cortex, *Cx3cl1*: 1.11 ± 0.14 control + Poly I:C vs. 0.74 ± 0.05 $MIA_{PPI\text{-}low}$ + Poly I:C, $F_{(2,33)}$ = 2.57, p = 0.0304 (Table 3)
- Frontal cortex, *Cx3cr1*: 1.01 ± 0.17 $MIA_{PPI\text{-}high}$ + vehicle vs. 0.67 ± 0.06 $MIA_{PPI\text{-}high}$ + Poly I:C, $F_{(5,34)}$ = 1.98, p = 0.0483 (Table 3)
- Hippocampus, *Cx3cl1*: 1.03 ± 0.08 control + vehicle vs. 0.70 ± 0.04 $MIA_{PPI\text{-}high}$ + vehicle, $F_{(5,37)}$ = 6.05, p = 0.0015; 0.94 ± 0.08 $MIA_{PPI\text{-}low}$ + vehicle vs. 0.70 ± 0.04 $MIA_{PPI\text{-}high}$ + vehicle, $F_{(2,37)}$ = 5.60, p = 0.0196; 1.20 ± 0.08 control + Poly I:C vs. 0.82 ± 0.03 $MIA_{PPI\text{-}high}$ + Poly I:C, $F_{(2,37)}$ = 6.41, p = 0.0011; 1.09 ± 0.12 $MIA_{PPI\text{-}low}$ + Poly I:C vs. 0.82 ± 0.03 $MIA_{PPI\text{-}high}$ + Poly I:C, $F_{(2,37)}$ = 5.60, p = 0.0278 (Table 3)
- Hippocampus, *Cx3cr1*: 1.05 ± 0.13 control + vehicle vs. 0.74 ± 0.04 $MIA_{PPI\text{-}high}$ + vehicle, $F_{(5,37)}$ = 1.86, p = 0.0416; 1.02 ± 0.09 control + Poly I:C vs. 0.76 ± 0.06 $MIA_{PPI\text{-}high}$ + Poly I:C, $F_{(2,37)}$ = 2.16, p = 0.0489 (Table 3)

2.8. Levels of CX3CL1, CX3CR1, CD200 and CD200R proteins in the frontal cortices and hippocampi of adult male offspring

- Frontal cortex, CX3CL1: 0.10 ± 0.01 control + vehicle vs. 0.16 ± 0.02 $MIA_{PPI\text{-}high}$ + vehicle, $F_{(5,38)}$ = 4.73, p = 0.0223; 0.13 ± 0.02 $MIA_{PPI\text{-}low}$ + Poly I:C vs. 0.20 ± 0.02 $MIA_{PPI\text{-}high}$ + Poly I:C, $F_{(2,38)}$ = 5.09, p = 0.0065; 0.11 ± 0.02 control + Poly I:C vs. 0.20 ± 0.02 $MIA_{PPI\text{-}high}$ + Poly I:C, $F_{(2,38)}$ = 8.36, p = 0.0003 (Figure 7A)
- Frontal cortex, CX3CR1: 26.84 ± 1.34 control + vehicle vs. 33.95 ± 2.06 $MIA_{PPI\text{-}high}$ + vehicle, $F_{(5,38)}$ = 1.73, p = 0.0265 (Figure 7A)
- Hippocampus, CX3CL1: 0.09 ± 0.01 control + vehicle vs. 0.13 ± 0.02 control + Poly I:C, $F_{(5,38)}$ = 2.62, p = 0.0493; 0.10 ± 0.01 $MIA_{PPI\text{-}high}$ + vehicle vs. 0.15 ± 0.01 $MIA_{PPI\text{-}high}$ + Poly I:C, $F_{(5,38)}$ = 2.62, p = 0.0228 (Figure 7B)
- Hippocampus, CX3CR1: 15.09 ± 0.59 $MIA_{PPI\text{-}low}$ + Poly I:C vs. 12.41 ± 0.54 $MIA_{PPI\text{-}high}$ + Poly I:C, $F_{(2,38)}$ = 3.32, p = 0.0144; 14.40 ± 0.77 control + Poly I:C vs. 12.41 ± 0.54 $MIA_{PPI\text{-}high}$ + Poly I:C, $F_{(2,38)}$ = 3.72, p = 0.0418 (Figure 7B)
- Hippocampus, CD200: 75.67 ± 2.83 $MIA_{PPI\text{-}high}$ + vehicle vs. 67.21 ± 2.49 $MIA_{PPI\text{-}high}$ + Poly I:C, $F_{(5,38)}$ = 2.57, p = 0.0455; 80.43 ± 3.31 control + Poly I:C vs. 67.21 ± 2.49 $MIA_{PPI\text{-}high}$ + Poly I:C, $F_{(2,38)}$ = 5.91, p = 0.0015 (Figure 7B)
- Hippocampus, CD200R: 22.49 ± 0.95 $MIA_{PPI\text{-}high}$ + vehicle vs. 19.26 ± 0.66 $MIA_{PPI\text{-}high}$ + Poly I:C, $F_{(5,38)}$ = 1.78, p = 0.0418; 22.20 ± 1.26 control + Poly I:C vs. 19.26 ± 0.66 $MIA_{PPI\text{-}high}$ + Poly I:C, $F_{(2,38)}$ = 2.32, p = 0.0484 (Figure 7B)

2.9. IBA1 levels in the frontal cortices and hippocampi of adult male offspring

- Frontal cortex, IBA1: 0.032 ± 0.0034 control + Poly I:C vs. 0.043 ± 0.0023 $MIA_{PPI\text{-}low}$ + Poly I:C, $F_{(2,17)}$ = 5.99, p = 0.0047; 0.043 ± 0.0023 $MIA_{PPI\text{-}low}$ + Poly I:C vs. 0.035 ± 0.0019 $MIA_{PPI\text{-}high}$ + Poly I:C, $F_{(2,17)}$ = 5.40, p = 0.0201 (Figure 8A)
- Hippocampus, IBA1: 0.031 ± 0.0055 control + Poly I:C vs. 0.051 ± 0.0078 $MIA_{PPI\text{-}high}$ + Poly I:C, $F_{(2,17)}$ 2.71= , p = 0.0326 (Figure 8B)
